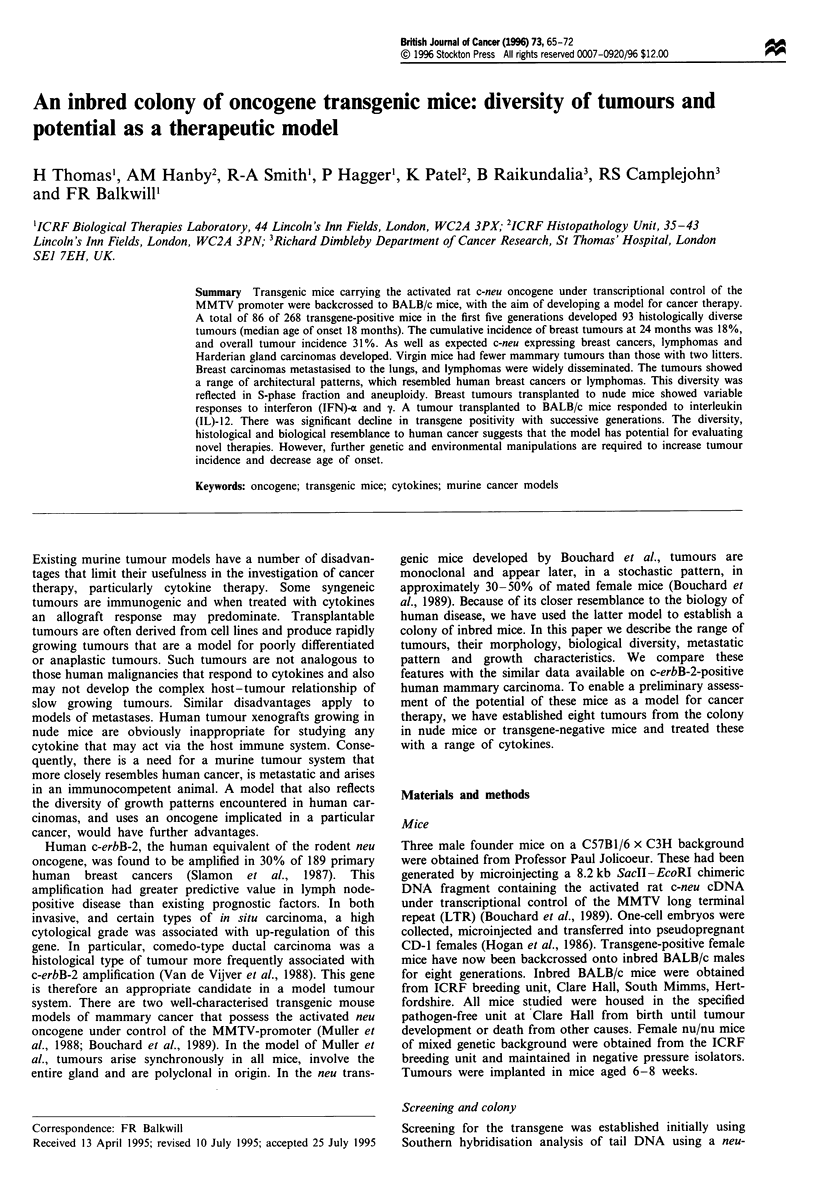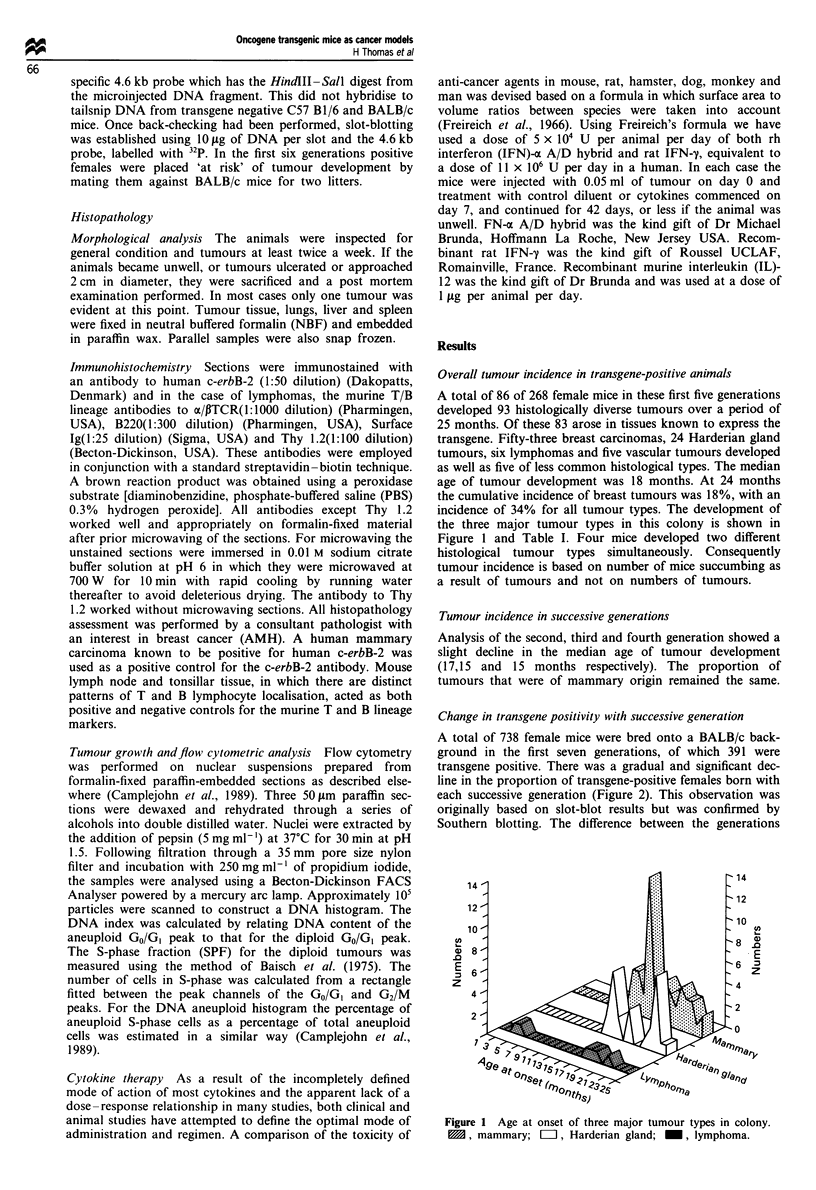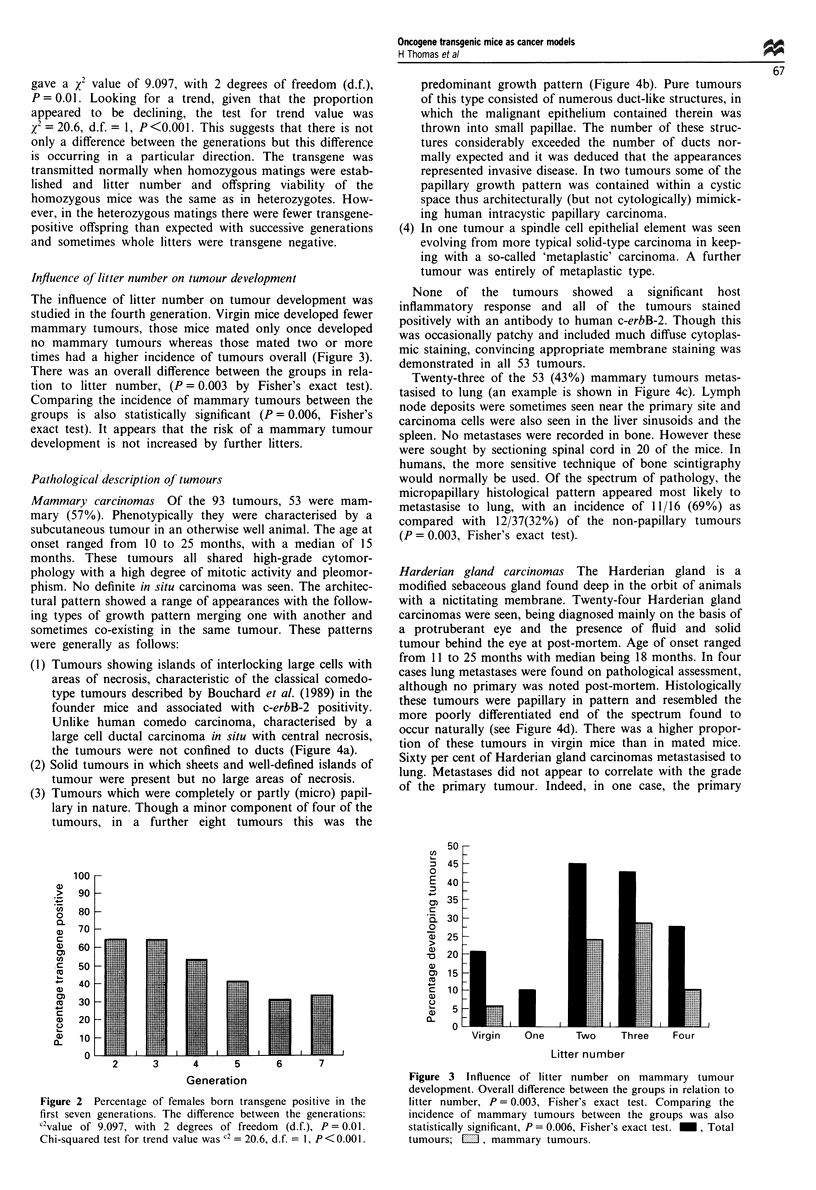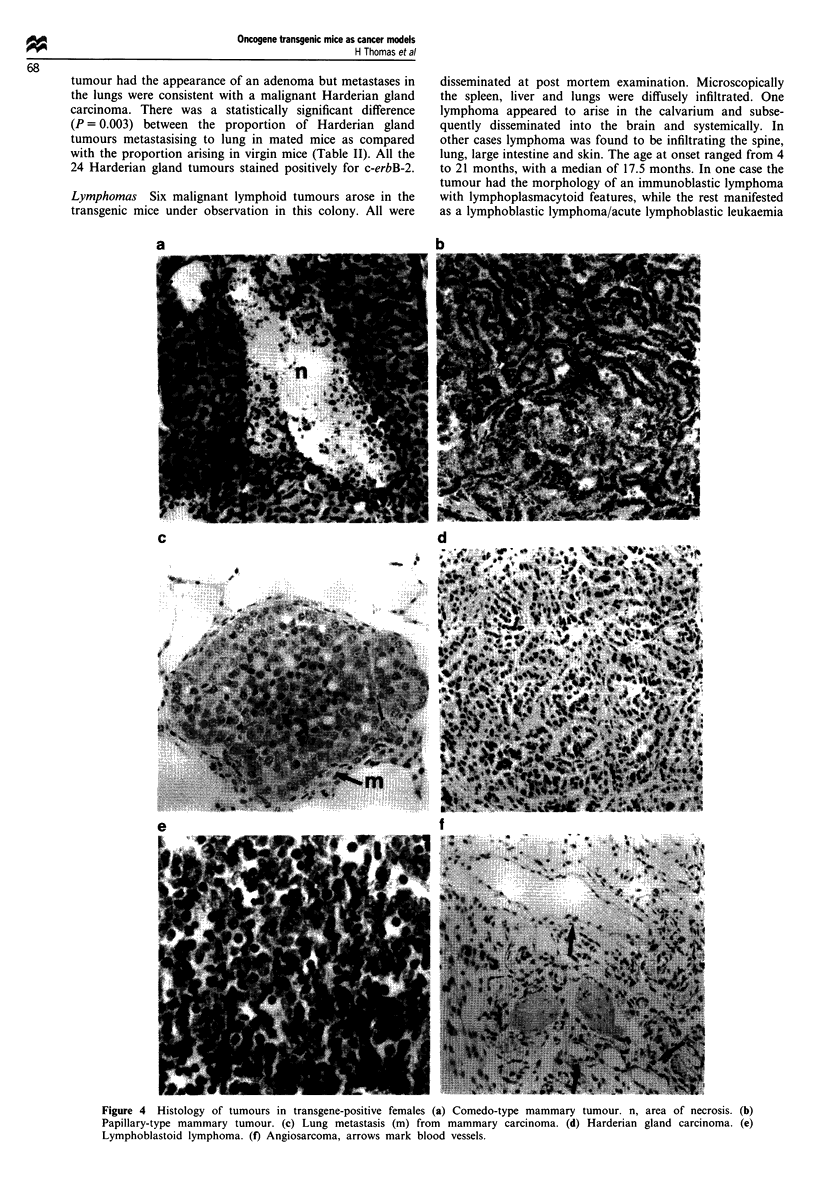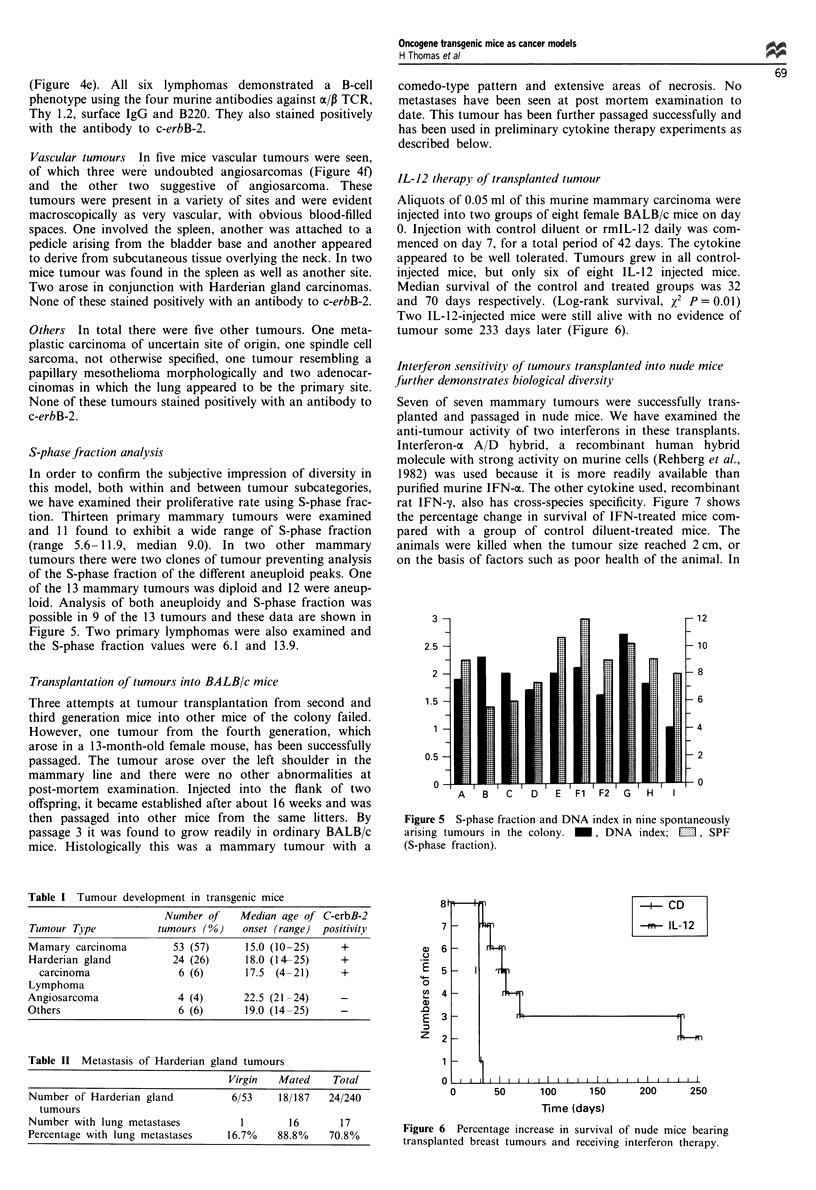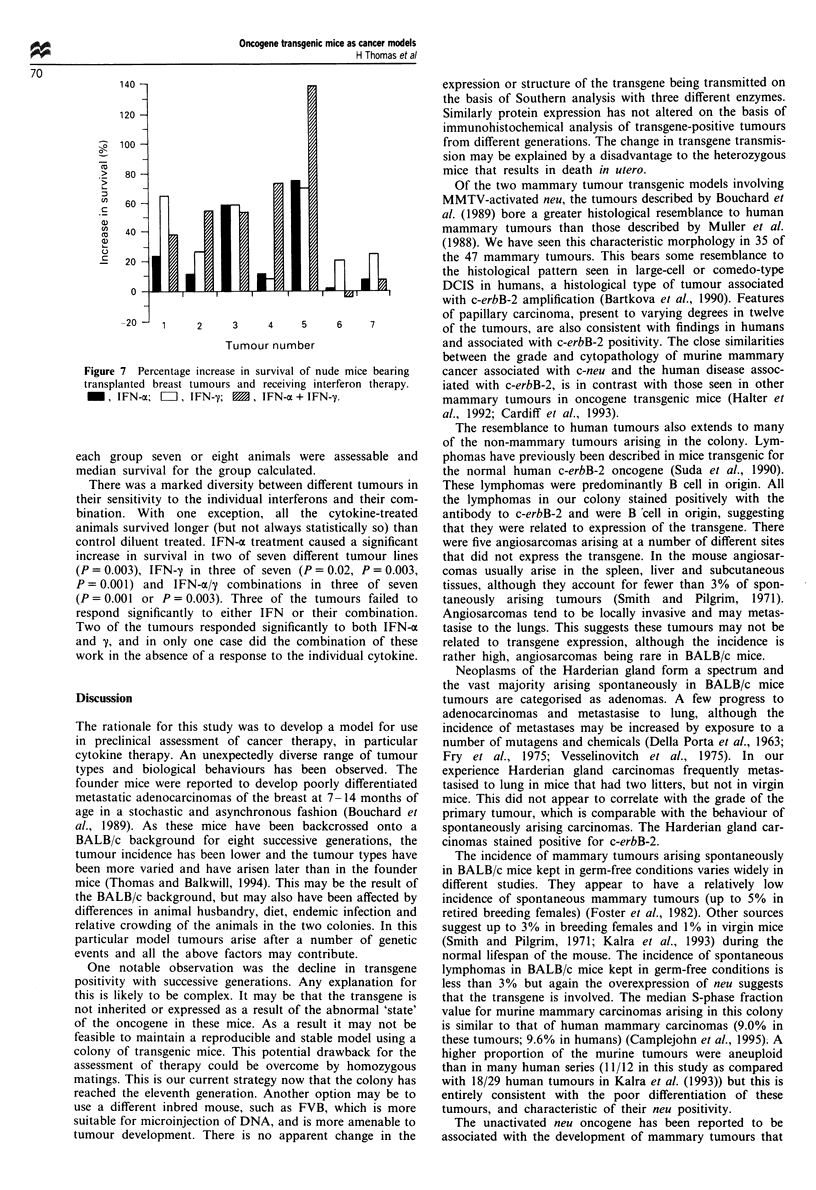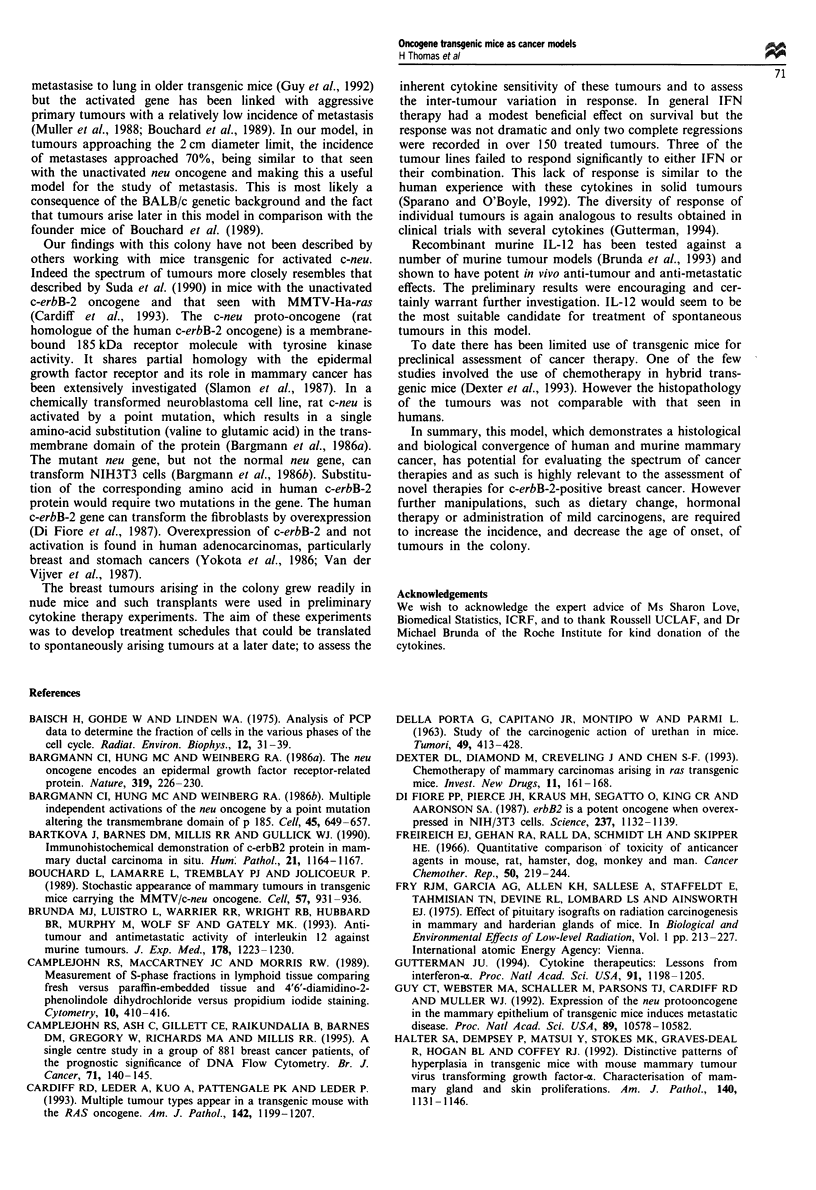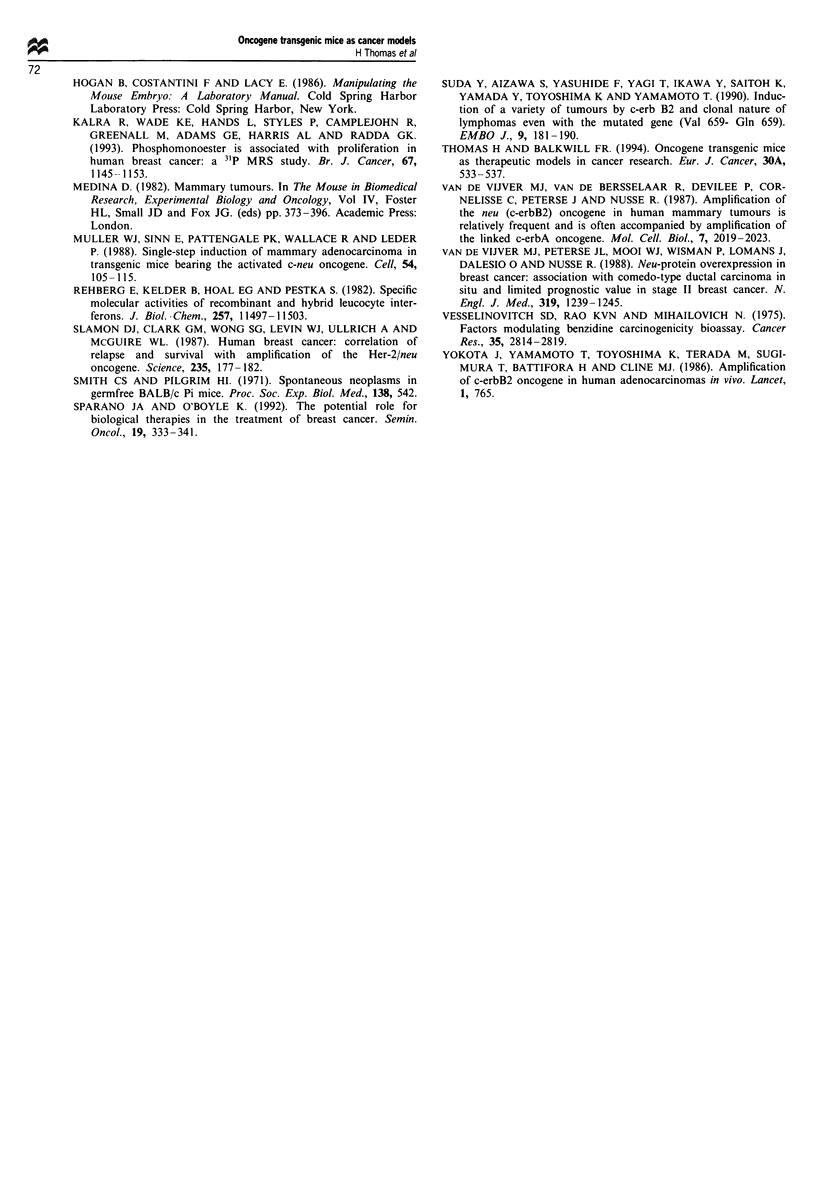# An inbred colony of oncogene transgenic mice: diversity of tumours and potential as a therapeutic model.

**DOI:** 10.1038/bjc.1996.12

**Published:** 1996-01

**Authors:** H. Thomas, A. M. Hanby, R. A. Smith, P. Hagger, K. Patel, B. Raikundalia, R. S. Camplejohn, F. R. Balkwill

**Affiliations:** ICRF Biological Therapies Laboratory, London, UK.

## Abstract

**Images:**


British Journal of Cancer (1996) 73, 65-72
© 1996 Stockton Press All rights reserved 0007-0920/96 $12.00

### An inbred colony of oncogene transgenic mice: diversity of tumours and potential as a therapeutic model

H Thomas[1], AM Hanby[2], R-A Smith[1], P Hagger[1], K Patel[2], B Raikundalia[3], RS Camplejohn[3] and FR Balkwill[1]

[1]*ICRF Biological Therapies Laboratory, 44 Lincoln's Inn Fields, London, WC2A 3PX;* [2]*ICRF Histopathology Unit, 35-43 Lincoln's Inn Fields, London, WC2A 3PN;* [3]*Richard Dimbleby Department of Cancer Research, St Thomas' Hospital, London SE1 7EH, UK.*

**Summary** Transgenic mice carrying the activated rat c-*neu* oncogene under transcriptional control of the MMTV promoter were backcrossed to BALB/c mice, with the aim of developing a model for cancer therapy. A total of 86 of 268 transgene-positive mice in the first five generations developed 93 histologically diverse tumours (median age of onset 18 months). The cumulative incidence of breast tumours at 24 months was 18%, and overall tumour incidence 31%. As well as expected c-*neu* expressing breast cancers, lymphomas and Harderian gland carcinomas developed. Virgin mice had fewer mammary tumours than those with two litters. Breast carcinomas metastasised to the lungs, and lymphomas were widely disseminated. The tumours showed a range of architectural patterns, which resembled human breast cancers or lymphomas. This diversity was reflected in S-phase fraction and aneuploidy. Breast tumours transplanted to nude mice showed variable responses to interferon (IFN)-α and γ. A tumour transplanted to BALB/c mice responded to interleukin (IL)-12. There was significant decline in transgene positivity with successive generations. The diversity, histological and biological resemblance to human cancer suggests that the model has potential for evaluating novel therapies. However, further genetic and environmental manipulations are required to increase tumour incidence and decrease age of onset.

**Keywords:** oncogene; transgenic mice; cytokines; murine cancer models

Existing murine tumour models have a number of disadvantages that limit their usefulness in the investigation of cancer therapy, particularly cytokine therapy. Some syngeneic tumours are immunogenic and when treated with cytokines an allograft response may predominate. Transplantable tumours are often derived from cell lines and produce rapidly growing tumours that are a model for poorly differentiated or anaplastic tumours. Such tumours are not analogous to those human malignancies that respond to cytokines and also may not develop the complex host-tumour relationship of slow growing tumours. Similar disadvantages apply to models of metastases. Human tumour xenografts growing in nude mice are obviously inappropriate for studying any cytokine that may act via the host immune system. Consequently, there is a need for a murine tumour system that more closely resembles human cancer, is metastatic and arises in an immunocompetent animal. A model that also reflects the diversity of growth patterns encountered in human carcinomas, and uses an oncogene implicated in a particular cancer, would have further advantages.

Human c-*erb*B-2, the human equivalent of the rodent *neu* oncogene, was found to be amplified in 30% of 189 primary human breast cancers (Slamon *et al.*, 1987). This amplification had greater predictive value in lymph node-positive disease than existing prognostic factors. In both invasive, and certain types of *in situ* carcinoma, a high cytological grade was associated with up-regulation of this gene. In particular, comedo-type ductal carcinoma was a histological type of tumour more frequently associated with c-*erb*B-2 amplification (Van de Vijver *et al.*, 1988). This gene is therefore an appropriate candidate in a model tumour system. There are two well-characterised transgenic mouse models of mammary cancer that possess the activated *neu* oncogene under control of the MMTV-promoter (Muller *et al.*, 1988; Bouchard *et al.*, 1989). In the model of Muller *et al.*, tumours arise synchronously in all mice, involve the entire gland and are polyclonal in origin. In the *neu* transgenic mice developed by Bouchard *et al.*, tumours are monoclonal and appear later, in a stochastic pattern, in approximately 30-50% of mated female mice (Bouchard *et al.*, 1989). Because of its closer resemblance to the biology of human disease, we have used the latter model to establish a colony of inbred mice. In this paper we describe the range of tumours, their morphology, biological diversity, metastatic pattern and growth characteristics. We compare these features with the similar data available on c-*erb*B-2-positive human mammary carcinoma. To enable a preliminary assessment of the potential of these mice as a model for cancer therapy, we have established eight tumours from the colony in nude mice or transgene-negative mice and treated these with a range of cytokines.

### Materials and methods

### *Mice*

Three male founder mice on a C57Bl/6 × C3H background were obtained from Professor Paul Jolicoeur. These had been generated by microinjecting a 8.2 kb *Sac*II-*Eco*RI chimeric DNA fragment containing the activated rat c-*neu* cDNA under transcriptional control of the MMTV long terminal repeat (LTR) (Bouchard *et al.*, 1989). One-cell embryos were collected, microinjected and transferred into pseudopregnant CD-1 females (Hogan *et al.*, 1986). Transgene-positive female mice have now been backcrossed onto inbred BALB/c males for eight generations. Inbred BALB/c mice were obtained from ICRF breeding unit, Clare Hall, South Mimms, Hertfordshire. All mice studied were housed in the specified pathogen-free unit at Clare Hall from birth until tumour development or death from other causes. Female nu/nu mice of mixed genetic background were obtained from the ICRF breeding unit and maintained in negative pressure isolators. Tumours were implanted in mice aged 6-8 weeks.

### *Screening and colony*

Screening for the transgene was established initially using Southern hybridisation analysis of tail DNA using a *neu*-

Correspondence: FR Balkwill
Received 13 April 1995; revised 10 July 1995; accepted 25 July 1995

specific 4.6 kb probe which has the *Hind*III–*Sal*I digest from the microinjected DNA fragment. This did not hybridise to tailsnip DNA from transgene negative C57 Bl/6 and BALB/c mice. Once back-checking had been performed, slot-blotting was established using 10 μg of DNA per slot and the 4.6 kb probe, labelled with $^{32}P$. In the first six generations positive females were placed 'at risk' of tumour development by mating them against BALB/c mice for two litters.

*Histopathology*

*Morphological analysis* The animals were inspected for general condition and tumours at least twice a week. If the animals became unwell, or tumours ulcerated or approached 2 cm in diameter, they were sacrificed and a post mortem examination performed. In most cases only one tumour was evident at this point. Tumour tissue, lungs, liver and spleen were fixed in neutral buffered formalin (NBF) and embedded in paraffin wax. Parallel samples were also snap frozen.

*Immunohistochemistry* Sections were immunostained with an antibody to human c-*erb*B-2 (1:50 dilution) (Dakopatts, Denmark) and in the case of lymphomas, the murine T/B lineage antibodies to α/βTCR(1:1000 dilution) (Pharmingen, USA), B220(1:300 dilution) (Pharmingen, USA), Surface Ig(1:25 dilution) (Sigma, USA) and Thy 1.2(1:100 dilution) (Becton-Dickinson, USA). These antibodies were employed in conjunction with a standard streptavidin–biotin technique. A brown reaction product was obtained using a peroxidase substrate [diaminobenzidine, phosphate-buffered saline (PBS) 0.3% hydrogen peroxide]. All antibodies except Thy 1.2 worked well and appropriately on formalin-fixed material after prior microwaving of the sections. For microwaving the unstained sections were immersed in 0.01 M sodium citrate buffer solution at pH 6 in which they were microwaved at 700 W for 10 min with rapid cooling by running water thereafter to avoid deleterious drying. The antibody to Thy 1.2 worked without microwaving sections. All histopathology assessment was performed by a consultant pathologist with an interest in breast cancer (AMH). A human mammary carcinoma known to be positive for human c-*erb*B-2 was used as a positive control for the c-*erb*B-2 antibody. Mouse lymph node and tonsillar tissue, in which there are distinct patterns of T and B lymphocyte localisation, acted as both positive and negative controls for the murine T and B lineage markers.

*Tumour growth and flow cytometric analysis* Flow cytometry was performed on nuclear suspensions prepared from formalin-fixed paraffin-embedded sections as described elsewhere (Camplejohn *et al*., 1989). Three 50 μm paraffin sections were dewaxed and rehydrated through a series of alcohols into double distilled water. Nuclei were extracted by the addition of pepsin (5 mg $ml^{-1}$) at 37°C for 30 min at pH 1.5. Following filtration through a 35 mm pore size nylon filter and incubation with 250 mg $ml^{-1}$ of propidium iodide, the samples were analysed using a Becton-Dickinson FACS Analyser powered by a mercury arc lamp. Approximately $10^5$ particles were scanned to construct a DNA histogram. The DNA index was calculated by relating DNA content of the aneuploid $G_0/G_1$ peak to that for the diploid $G_0/G_1$ peak. The S-phase fraction (SPF) for the diploid tumours was measured using the method of Baisch *et al*. (1975). The number of cells in S-phase was calculated from a rectangle fitted between the peak channels of the $G_0/G_1$ and $G_2/M$ peaks. For the DNA aneuploid histogram the percentage of aneuploid S-phase cells as a percentage of total aneuploid cells was estimated in a similar way (Camplejohn *et al*., 1989).

*Cytokine therapy* As a result of the incompletely defined mode of action of most cytokines and the apparent lack of a dose–response relationship in many studies, both clinical and animal studies have attempted to define the optimal mode of administration and regimen. A comparison of the toxicity of anti-cancer agents in mouse, rat, hamster, dog, monkey and man was devised based on a formula in which surface area to volume ratios between species were taken into account (Freireich *et al*., 1966). Using Freireich's formula we have used a dose of $5 \times 10^4$ U per animal per day of both rh interferon (IFN)-α A/D hybrid and rat IFN-γ, equivalent to a dose of $11 \times 10^6$ U per day in a human. In each case the mice were injected with 0.05 ml of tumour on day 0 and treatment with control diluent or cytokines commenced on day 7, and continued for 42 days, or less if the animal was unwell. FN-α A/D hybrid was the kind gift of Dr Michael Brunda, Hoffmann La Roche, New Jersey USA. Recombinant rat IFN-γ was the kind gift of Roussel UCLAF, Romainville, France. Recombinant murine interleukin (IL)-12 was the kind gift of Dr Brunda and was used at a dose of 1 μg per animal per day.

### Results

*Overall tumour incidence in transgene-positive animals*

A total of 86 of 268 female mice in these first five generations developed 93 histologically diverse tumours over a period of 25 months. Of these 83 arose in tissues known to express the transgene. Fifty-three breast carcinomas, 24 Harderian gland tumours, six lymphomas and five vascular tumours developed as well as five of less common histological types. The median age of tumour development was 18 months. At 24 months the cumulative incidence of breast tumours was 18%, with an incidence of 34% for all tumour types. The development of the three major tumour types in this colony is shown in Figure 1 and Table I. Four mice developed two different histological tumour types simultaneously. Consequently tumour incidence is based on number of mice succumbing as a result of tumours and not on numbers of tumours.

*Tumour incidence in successive generations*

Analysis of the second, third and fourth generation showed a slight decline in the median age of tumour development (17,15 and 15 months respectively). The proportion of tumours that were of mammary origin remained the same.

*Change in transgene positivity with successive generation*

A total of 738 female mice were bred onto a BALB/c background in the first seven generations, of which 391 were transgene positive. There was a gradual and significant decline in the proportion of transgene-positive females born with each successive generation (Figure 2). This observation was originally based on slot-blot results but was confirmed by Southern blotting. The difference between the generations

**Figure 1** Age at onset of three major tumour types in colony. ▨, mammary; ▭, Harderian gland; ■, lymphoma.

gave a $\chi^2$ value of 9.097, with 2 degrees of freedom (d.f.), $P = 0.01$. Looking for a trend, given that the proportion appeared to be declining, the test for trend value was $\chi^2 = 20.6$, d.f. = 1, $P < 0.001$. This suggests that there is not only a difference between the generations but this difference is occurring in a particular direction. The transgene was transmitted normally when homozygous matings were established and litter number and offspring viability of the homozygous mice was the same as in heterozygotes. However, in the heterozygous matings there were fewer transgene-positive offspring than expected with successive generations and sometimes whole litters were transgene negative.

### *Influence of litter number on tumour development*

The influence of litter number on tumour development was studied in the fourth generation. Virgin mice developed fewer mammary tumours, those mice mated only once developed no mammary tumours whereas those mated two or more times had a higher incidence of tumours overall (Figure 3). There was an overall difference between the groups in relation to litter number, ($P = 0.003$ by Fisher's exact test). Comparing the incidence of mammary tumours between the groups is also statistically significant ($P = 0.006$, Fisher's exact test). It appears that the risk of a mammary tumour development is not increased by further litters.

### *Pathological description of tumours*

*Mammary carcinomas* Of the 93 tumours, 53 were mammary (57%). Phenotypically they were characterised by a subcutaneous tumour in an otherwise well animal. The age at onset ranged from 10 to 25 months, with a median of 15 months. These tumours all shared high-grade cytomorphology with a high degree of mitotic activity and pleomorphism. No definite *in situ* carcinoma was seen. The architectural pattern showed a range of appearances with the following types of growth pattern merging one with another and sometimes co-existing in the same tumour. These patterns were generally as follows:

(1) Tumours showing islands of interlocking large cells with areas of necrosis, characteristic of the classical comedo-type tumours described by Bouchard *et al.* (1989) in the founder mice and associated with c-*erb*B-2 positivity. Unlike human comedo carcinoma, characterised by a large cell ductal carcinoma *in situ* with central necrosis, the tumours were not confined to ducts (Figure 4a).
(2) Solid tumours in which sheets and well-defined islands of tumour were present but no large areas of necrosis.
(3) Tumours which were completely or partly (micro) papillary in nature. Though a minor component of four of the tumours, in a further eight tumours this was the predominant growth pattern (Figure 4b). Pure tumours of this type consisted of numerous duct-like structures, in which the malignant epithelium contained therein was thrown into small papillae. The number of these structures considerably exceeded the number of ducts normally expected and it was deduced that the appearances represented invasive disease. In two tumours some of the papillary growth pattern was contained within a cystic space thus architecturally (but not cytologically) mimicking human intracystic papillary carcinoma.
(4) In one tumour a spindle cell epithelial element was seen evolving from more typical solid-type carcinoma in keeping with a so-called 'metaplastic' carcinoma. A further tumour was entirely of metaplastic type.

None of the tumours showed a significant host inflammatory response and all of the tumours stained positively with an antibody to human c-*erb*B-2. Though this was occasionally patchy and included much diffuse cytoplasmic staining, convincing appropriate membrane staining was demonstrated in all 53 tumours.

Twenty-three of the 53 (43%) mammary tumours metastasised to lung (an example is shown in Figure 4c). Lymph node deposits were sometimes seen near the primary site and carcinoma cells were also seen in the liver sinusoids and the spleen. No metastases were recorded in bone. However these were sought by sectioning spinal cord in 20 of the mice. In humans, the more sensitive technique of bone scintigraphy would normally be used. Of the spectrum of pathology, the micropapillary histological pattern appeared most likely to metastasise to lung, with an incidence of 11/16 (69%) as compared with 12/37(32%) of the non-papillary tumours ($P = 0.003$, Fisher's exact test).

*Harderian gland carcinomas* The Harderian gland is a modified sebaceous gland found deep in the orbit of animals with a nictitating membrane. Twenty-four Harderian gland carcinomas were seen, being diagnosed mainly on the basis of a protruberant eye and the presence of fluid and solid tumour behind the eye at post-mortem. Age of onset ranged from 11 to 25 months with median being 18 months. In four cases lung metastases were found on pathological assessment, although no primary was noted post-mortem. Histologically these tumours were papillary in pattern and resembled the more poorly differentiated end of the spectrum found to occur naturally (see Figure 4d). There was a higher proportion of these tumours in virgin mice than in mated mice. Sixty per cent of Harderian gland carcinomas metastasised to lung. Metastases did not appear to correlate with the grade of the primary tumour. Indeed, in one case, the primary

**Figure 2** Percentage of females born transgene positive in the first seven generations. The difference between the generations: $^{c2}$value of 9.097, with 2 degrees of freedom (d.f.), $P = 0.01$. Chi-squared test for trend value was $^{c2} = 20.6$, d.f. = 1, $P < 0.001$.

**Figure 3** Influence of litter number on mammary tumour development. Overall difference between the groups in relation to litter number, $P = 0.003$, Fisher's exact test. Comparing the incidence of mammary tumours between the groups was also statistically significant, $P = 0.006$, Fisher's exact test. ■, Total tumours; ▨, mammary tumours.

tumour had the appearance of an adenoma but metastases in the lungs were consistent with a malignant Harderian gland carcinoma. There was a statistically significant difference ($P = 0.003$) between the proportion of Harderian gland tumours metastasising to lung in mated mice as compared with the proportion arising in virgin mice (Table II). All the 24 Harderian gland tumours stained positively for c-*erb*B-2.

*Lymphomas* Six malignant lymphoid tumours arose in the transgenic mice under observation in this colony. All were disseminated at post mortem examination. Microscopically the spleen, liver and lungs were diffusely infiltrated. One lymphoma appeared to arise in the calvarium and subsequently disseminated into the brain and systemically. In other cases lymphoma was found to be infiltrating the spine, lung, large intestine and skin. The age at onset ranged from 4 to 21 months, with a median of 17.5 months. In one case the tumour had the morphology of an immunoblastic lymphoma with lymphoplasmacytoid features, while the rest manifested as a lymphoblastic lymphoma/acute lymphoblastic leukaemia

**Figure 4** Histology of tumours in transgene-positive females (**a**) Comedo-type mammary tumour. n, area of necrosis. (**b**) Papillary-type mammary tumour. (**c**) Lung metastasis (m) from mammary carcinoma. (**d**) Harderian gland carcinoma. (**e**) Lymphoblastoid lymphoma. (**f**) Angiosarcoma, arrows mark blood vessels.

(Figure 4e). All six lymphomas demonstrated a B-cell phenotype using the four murine antibodies against α/β TCR, Thy 1.2, surface IgG and B220. They also stained positively with the antibody to c-*erb*B-2.

*Vascular tumours* In five mice vascular tumours were seen, of which three were undoubted angiosarcomas (Figure 4f) and the other two suggestive of angiosarcoma. These tumours were present in a variety of sites and were evident macroscopically as very vascular, with obvious blood-filled spaces. One involved the spleen, another was attached to a pedicle arising from the bladder base and another appeared to derive from subcutaneous tissue overlying the neck. In two mice tumour was found in the spleen as well as another site. Two arose in conjunction with Harderian gland carcinomas. None of these stained positively with an antibody to c-*erb*B-2.

*Others* In total there were five other tumours. One metaplastic carcinoma of uncertain site of origin, one spindle cell sarcoma, not otherwise specified, one tumour resembling a papillary mesothelioma morphologically and two adenocarcinomas in which the lung appeared to be the primary site. None of these tumours stained positively with an antibody to c-*erb*B-2.

### *S-phase fraction analysis*

In order to confirm the subjective impression of diversity in this model, both within and between tumour subcategories, we have examined their proliferative rate using S-phase fraction. Thirteen primary mammary tumours were examined and 11 found to exhibit a wide range of S-phase fraction (range 5.6–11.9, median 9.0). In two other mammary tumours there were two clones of tumour preventing analysis of the S-phase fraction of the different aneuploid peaks. One of the 13 mammary tumours was diploid and 12 were aneuploid. Analysis of both aneuploidy and S-phase fraction was possible in 9 of the 13 tumours and these data are shown in Figure 5. Two primary lymphomas were also examined and the S-phase fraction values were 6.1 and 13.9.

### *Transplantation of tumours into BALB/c mice*

Three attempts at tumour transplantation from second and third generation mice into other mice of the colony failed. However, one tumour from the fourth generation, which arose in a 13-month-old female mouse, has been successfully passaged. The tumour arose over the left shoulder in the mammary line and there were no other abnormalities at post-mortem examination. Injected into the flank of two offspring, it became established after about 16 weeks and was then passaged into other mice from the same litters. By passage 3 it was found to grow readily in ordinary BALB/c mice. Histologically this was a mammary tumour with a comedo-type pattern and extensive areas of necrosis. No metastases have been seen at post mortem examination to date. This tumour has been further passaged successfully and has been used in preliminary cytokine therapy experiments as described below.

### *IL-12 therapy of transplanted tumour*

Aliquots of 0.05 ml of this murine mammary carcinoma were injected into two groups of eight female BALB/c mice on day 0. Injection with control diluent or rmIL-12 daily was commenced on day 7, for a total period of 42 days. The cytokine appeared to be well tolerated. Tumours grew in all control-injected mice, but only six of eight IL-12 injected mice. Median survival of the control and treated groups was 32 and 70 days respectively. (Log-rank survival, $\chi^2$ $P = 0.01$) Two IL-12-injected mice were still alive with no evidence of tumour some 233 days later (Figure 6).

### *Interferon sensitivity of tumours transplanted into nude mice further demonstrates biological diversity*

Seven of seven mammary tumours were successfully transplanted and passaged in nude mice. We have examined the anti-tumour activity of two interferons in these transplants. Interferon-α A/D hybrid, a recombinant human hybrid molecule with strong activity on murine cells (Rehberg *et al.*, 1982) was used because it is more readily available than purified murine IFN-α. The other cytokine used, recombinant rat IFN-γ, also has cross-species specificity. Figure 7 shows the percentage change in survival of IFN-treated mice compared with a group of control diluent-treated mice. The animals were killed when the tumour size reached 2 cm, or on the basis of factors such as poor health of the animal. In

**Figure 5** S-phase fraction and DNA index in nine spontaneously arising tumours in the colony. ■, DNA index; ▒, SPF (S-phase fraction).

**Figure 6** Percentage increase in survival of nude mice bearing transplanted breast tumours and receiving interferon therapy.

**Table I** Tumour development in transgenic mice

| *Tumour Type* | *Number of tumours (%)* | *Median age of onset (range)* | *C-*erb*B-2 positivity* |
|---|---|---|---|
| Mamary carcinoma | 53 (57) | 15.0 (10-25) | + |
| Harderian gland carcinoma | 24 (26) | 18.0 (14-25) | + |
| Lymphoma | 6 (6) | 17.5 (4-21) | + |
| Angiosarcoma | 4 (4) | 22.5 (21-24) | – |
| Others | 6 (6) | 19.0 (14-25) | – |

**Table II** Metastasis of Harderian gland tumours

| | *Virgin* | *Mated* | *Total* |
|---|---|---|---|
| Number of Harderian gland tumours | 6/53 | 18/187 | 24/240 |
| Number with lung metastases | 1 | 16 | 17 |
| Percentage with lung metastases | 16.7% | 88.8% | 70.8% |

**Figure 7** Percentage increase in survival of nude mice bearing transplanted breast tumours and receiving interferon therapy. ■, IFN-α; □, IFN-γ; ▨, IFN-α + IFN-γ.

each group seven or eight animals were assessable and median survival for the group calculated.

There was a marked diversity between different tumours in their sensitivity to the individual interferons and their combination. With one exception, all the cytokine-treated animals survived longer (but not always statistically so) than control diluent treated. IFN-α treatment caused a significant increase in survival in two of seven different tumour lines ($P = 0.003$), IFN-γ in three of seven ($P = 0.02$, $P = 0.003$, $P = 0.001$) and IFN-α/γ combinations in three of seven ($P = 0.001$ or $P = 0.003$). Three of the tumours failed to respond significantly to either IFN or their combination. Two of the tumours responded significantly to both IFN-α and γ, and in only one case did the combination of these work in the absence of a response to the individual cytokine.

### Discussion

The rationale for this study was to develop a model for use in preclinical assessment of cancer therapy, in particular cytokine therapy. An unexpectedly diverse range of tumour types and biological behaviours has been observed. The founder mice were reported to develop poorly differentiated metastatic adenocarcinomas of the breast at 7–14 months of age in a stochastic and asynchronous fashion (Bouchard *et al.*, 1989). As these mice have been backcrossed onto a BALB/c background for eight successive generations, the tumour incidence has been lower and the tumour types have been more varied and have arisen later than in the founder mice (Thomas and Balkwill, 1994). This may be the result of the BALB/c background, but may also have been affected by differences in animal husbandry, diet, endemic infection and relative crowding of the animals in the two colonies. In this particular model tumours arise after a number of genetic events and all the above factors may contribute.

One notable observation was the decline in transgene positivity with successive generations. Any explanation for this is likely to be complex. It may be that the transgene is not inherited or expressed as a result of the abnormal 'state' of the oncogene in these mice. As a result it may not be feasible to maintain a reproducible and stable model using a colony of transgenic mice. This potential drawback for the assessment of therapy could be overcome by homozygous matings. This is our current strategy now that the colony has reached the eleventh generation. Another option may be to use a different inbred mouse, such as FVB, which is more suitable for microinjection of DNA, and is more amenable to tumour development. There is no apparent change in the expression or structure of the transgene being transmitted on the basis of Southern analysis with three different enzymes. Similarly protein expression has not altered on the basis of immunohistochemical analysis of transgene-positive tumours from different generations. The change in transgene transmission may be explained by a disadvantage to the heterozygous mice that results in death *in utero*.

Of the two mammary tumour transgenic models involving MMTV-activated *neu*, the tumours described by Bouchard *et al.* (1989) bore a greater histological resemblance to human mammary tumours than those described by Muller *et al.* (1988). We have seen this characteristic morphology in 35 of the 47 mammary tumours. This bears some resemblance to the histological pattern seen in large-cell or comedo-type DCIS in humans, a histological type of tumour associated with c-*erb*B-2 amplification (Bartkova *et al.*, 1990). Features of papillary carcinoma, present to varying degrees in twelve of the tumours, are also consistent with findings in humans and associated with c-*erb*B-2 positivity. The close similarities between the grade and cytopathology of murine mammary cancer associated with c-*neu* and the human disease associated with c-*erb*B-2, is in contrast with those seen in other mammary tumours in oncogene transgenic mice (Halter *et al.*, 1992; Cardiff *et al.*, 1993).

The resemblance to human tumours also extends to many of the non-mammary tumours arising in the colony. Lymphomas have previously been described in mice transgenic for the normal human c-*erb*B-2 oncogene (Suda *et al.*, 1990). These lymphomas were predominantly B cell in origin. All the lymphomas in our colony stained positively with the antibody to c-*erb*B-2 and were B cell in origin, suggesting that they were related to expression of the transgene. There were five angiosarcomas arising at a number of different sites that did not express the transgene. In the mouse angiosarcomas usually arise in the spleen, liver and subcutaneous tissues, although they account for fewer than 3% of spontaneously arising tumours (Smith and Pilgrim, 1971). Angiosarcomas tend to be locally invasive and may metastasise to the lungs. This suggests these tumours may not be related to transgene expression, although the incidence is rather high, angiosarcomas being rare in BALB/c mice.

Neoplasms of the Harderian gland form a spectrum and the vast majority arising spontaneously in BALB/c mice tumours are categorised as adenomas. A few progress to adenocarcinomas and metastasise to lung, although the incidence of metastases may be increased by exposure to a number of mutagens and chemicals (Della Porta *et al.*, 1963; Fry *et al.*, 1975; Vesselinovitch *et al.*, 1975). In our experience Harderian gland carcinomas frequently metastasised to lung in mice that had two litters, but not in virgin mice. This did not appear to correlate with the grade of the primary tumour, which is comparable with the behaviour of spontaneously arising carcinomas. The Harderian gland carcinomas stained positive for c-*erb*B-2.

The incidence of mammary tumours arising spontaneously in BALB/c mice kept in germ-free conditions varies widely in different studies. They appear to have a relatively low incidence of spontaneous mammary tumours (up to 5% in retired breeding females) (Foster *et al.*, 1982). Other sources suggest up to 3% in breeding females and 1% in virgin mice (Smith and Pilgrim, 1971; Kalra *et al.*, 1993) during the normal lifespan of the mouse. The incidence of spontaneous lymphomas in BALB/c mice kept in germ-free conditions is less than 3% but again the overexpression of *neu* suggests that the transgene is involved. The median S-phase fraction value for murine mammary carcinomas arising in this colony is similar to that of human mammary carcinomas (9.0% in these tumours; 9.6% in humans) (Camplejohn *et al.*, 1995). A higher proportion of the murine tumours were aneuploid than in many human series (11/12 in this study as compared with 18/29 human tumours in Kalra *et al.* (1993)) but this is entirely consistent with the poor differentiation of these tumours, and characteristic of their *neu* positivity.

The unactivated *neu* oncogene has been reported to be associated with the development of mammary tumours that metastasise to lung in older transgenic mice (Guy et al., 1992) but the activated gene has been linked with aggressive primary tumours with a relatively low incidence of metastasis (Muller et al., 1988; Bouchard et al., 1989). In our model, in tumours approaching the 2 cm diameter limit, the incidence of metastases approached 70%, being similar to that seen with the unactivated *neu* oncogene and making this a useful model for the study of metastasis. This is most likely a consequence of the BALB/c genetic background and the fact that tumours arise later in this model in comparison with the founder mice of Bouchard et al. (1989).

Our findings with this colony have not been described by others working with mice transgenic for activated c-*neu*. Indeed the spectrum of tumours more closely resembles that described by Suda et al. (1990) in mice with the unactivated c-*erb*B-2 oncogene and that seen with MMTV-Ha-*ras* (Cardiff et al., 1993). The c-*neu* proto-oncogene (rat homologue of the human c-*erb*B-2 oncogene) is a membrane-bound 185 kDa receptor molecule with tyrosine kinase activity. It shares partial homology with the epidermal growth factor receptor and its role in mammary cancer has been extensively investigated (Slamon et al., 1987). In a chemically transformed neuroblastoma cell line, rat c-*neu* is activated by a point mutation, which results in a single amino-acid substitution (valine to glutamic acid) in the transmembrane domain of the protein (Bargmann et al., 1986*a*). The mutant *neu* gene, but not the normal *neu* gene, can transform NIH3T3 cells (Bargmann et al., 1986*b*). Substitution of the corresponding amino acid in human c-*erb*B-2 protein would require two mutations in the gene. The human c-*erb*B-2 gene can transform the fibroblasts by overexpression (Di Fiore et al., 1987). Overexpression of c-*erb*B-2 and not activation is found in human adenocarcinomas, particularly breast and stomach cancers (Yokota et al., 1986; Van der Vijver et al., 1987).

The breast tumours arising in the colony grew readily in nude mice and such transplants were used in preliminary cytokine therapy experiments. The aim of these experiments was to develop treatment schedules that could be translated to spontaneously arising tumours at a later date; to assess the inherent cytokine sensitivity of these tumours and to assess the inter-tumour variation in response. In general IFN therapy had a modest beneficial effect on survival but the response was not dramatic and only two complete regressions were recorded in over 150 treated tumours. Three of the tumour lines failed to respond significantly to either IFN or their combination. This lack of response is similar to the human experience with these cytokines in solid tumours (Sparano and O'Boyle, 1992). The diversity of response of individual tumours is again analogous to results obtained in clinical trials with several cytokines (Gutterman, 1994).

Recombinant murine IL-12 has been tested against a number of murine tumour models (Brunda et al., 1993) and shown to have potent *in vivo* anti-tumour and anti-metastatic effects. The preliminary results were encouraging and certainly warrant further investigation. IL-12 would seem to be the most suitable candidate for treatment of spontaneous tumours in this model.

To date there has been limited use of transgenic mice for preclinical assessment of cancer therapy. One of the few studies involved the use of chemotherapy in hybrid transgenic mice (Dexter et al., 1993). However the histopathology of the tumours was not comparable with that seen in humans.

In summary, this model, which demonstrates a histological and biological convergence of human and murine mammary cancer, has potential for evaluating the spectrum of cancer therapies and as such is highly relevant to the assessment of novel therapies for c-*erb*B-2-positive breast cancer. However further manipulations, such as dietary change, hormonal therapy or administration of mild carcinogens, are required to increase the incidence, and decrease the age of onset, of tumours in the colony.

**Acknowledgements**
We wish to acknowledge the expert advice of Ms Sharon Love, Biomedical Statistics, ICRF, and to thank Roussell UCLAF, and Dr Michael Brunda of the Roche Institute for kind donation of the cytokines.

### References

BAISCH H, GOHDE W AND LINDEN WA. (1975). Analysis of PCP data to determine the fraction of cells in the various phases of the cell cycle. *Radiat. Environ. Biophys.*, **12**, 31–39.

BARGMANN CI, HUNG MC AND WEINBERG RA. (1986*a*). The *neu* oncogene encodes an epidermal growth factor receptor-related protein. *Nature*, **319**, 226–230.

BARGMANN CI, HUNG MC AND WEINBERG RA. (1986*b*). Multiple independent activations of the *neu* oncogene by a point mutation altering the transmembrane domain of p 185. *Cell*, **45**, 649–657.

BARTKOVA J, BARNES DM, MILLIS RR AND GULLICK WJ. (1990). Immunohistochemical demonstration of c-erbB2 protein in mammary ductal carcinoma in situ. *Hum. Pathol.*, **21**, 1164–1167.

BOUCHARD L, LAMARRE L, TREMBLAY PJ AND JOLICOEUR P. (1989). Stochastic appearance of mammary tumours in transgenic mice carrying the MMTV/c-*neu* oncogene. *Cell*, **57**, 931–936.

BRUNDA MJ, LUISTRO L, WARRIER RR, WRIGHT RB, HUBBARD BR, MURPHY M, WOLF SF AND GATELY MK. (1993). Antitumour and antimetastatic activity of interleukin 12 against murine tumours. *J. Exp. Med.*, **178**, 1223–1230.

CAMPLEJOHN RS, MACCARTNEY JC AND MORRIS RW. (1989). Measurement of S-phase fractions in lymphoid tissue comparing fresh versus paraffin-embedded tissue and 4′6′-diamidino-2-phenolindole dihydrochloride versus propidium iodide staining. *Cytometry*, **10**, 410–416.

CAMPLEJOHN RS, ASH C, GILLETT CE, RAIKUNDALIA B, BARNES DM, GREGORY W, RICHARDS MA AND MILLIS RR. (1995). A single centre study in a group of 881 breast cancer patients, of the prognostic significance of DNA Flow Cytometry. *Br. J. Cancer*, **71**, 140–145.

CARDIFF RD, LEDER A, KUO A, PATTENGALE PK AND LEDER P. (1993). Multiple tumour types appear in a transgenic mouse with the *RAS* oncogene. *Am. J. Pathol.*, **142**, 1199–1207.

DELLA PORTA G, CAPITANO JR, MONTIPO W AND PARMI L. (1963). Study of the carcinogenic action of urethan in mice. *Tumori*, **49**, 413–428.

DEXTER DL, DIAMOND M, CREVELING J AND CHEN S-F. (1993). Chemotherapy of mammary carcinomas arising in *ras* transgenic mice. *Invest. New Drugs*, **11**, 161–168.

DI FIORE PP, PIERCE JH, KRAUS MH, SEGATTO O, KING CR AND AARONSON SA. (1987). *erbB2* is a potent oncogene when overexpressed in NIH/3T3 cells. *Science*, **237**, 1132–1139.

FREIREICH EJ, GEHAN RA, RALL DA, SCHMIDT LH AND SKIPPER HE. (1966). Quantitative comparison of toxicity of anticancer agents in mouse, rat, hamster, dog, monkey and man. *Cancer Chemother. Rep.*, **50**, 219–244.

FRY RJM, GARCIA AG, ALLEN KH, SALLESE A, STAFFELDT E, TAHMISIAN TN, DEVINE RL, LOMBARD LS AND AINSWORTH EJ. (1975). Effect of pituitary isografts on radiation carcinogenesis in mammary and harderian glands of mice. In *Biological and Environmental Effects of Low-level Radiation*, Vol. 1 pp. 213–227. International atomic Energy Agency: Vienna.

GUTTERMAN JU. (1994). Cytokine therapeutics: Lessons from interferon-α. *Proc. Natl Acad. Sci. USA*, **91**, 1198–1205.

GUY CT, WEBSTER MA, SCHALLER M, PARSONS TJ, CARDIFF RD AND MULLER WJ. (1992). Expression of the *neu* protooncogene in the mammary epithelium of transgenic mice induces metastatic disease. *Proc. Natl Acad. Sci. USA*, **89**, 10578–10582.

HALTER SA, DEMPSEY P, MATSUI Y, STOKES MK, GRAVES-DEAL R, HOGAN BL AND COFFEY RJ. (1992). Distinctive patterns of hyperplasia in transgenic mice with mouse mammary tumour virus transforming growth factor-α. Characterisation of mammary gland and skin proliferations. *Am. J. Pathol.*, **140**, 1131–1146.

HOGAN B, COSTANTINI F AND LACY E. (1986). *Manipulating the Mouse Embryo: A Laboratory Manual.* Cold Spring Harbor Laboratory Press: Cold Spring Harbor, New York.

KALRA R, WADE KE, HANDS L, STYLES P, CAMPLEJOHN R, GREENALL M, ADAMS GE, HARRIS AL AND RADDA GK. (1993). Phosphomonoester is associated with proliferation in human breast cancer: a $^{31}$P MRS study. *Br. J. Cancer*, **67,** 1145–1153.

MEDINA D. (1982). Mammary tumours. In *The Mouse in Biomedical Research, Experimental Biology and Oncology*, Vol IV, Foster HL, Small JD and Fox JG. (eds) pp. 373–396. Academic Press: London.

MULLER WJ, SINN E, PATTENGALE PK, WALLACE R AND LEDER P. (1988). Single-step induction of mammary adenocarcinoma in transgenic mice bearing the activated c-*neu* oncogene. *Cell*, **54,** 105–115.

REHBERG E, KELDER B, HOAL EG AND PESTKA S. (1982). Specific molecular activities of recombinant and hybrid leucocyte interferons. *J. Biol. Chem.*, **257,** 11497–11503.

SLAMON DJ, CLARK GM, WONG SG, LEVIN WJ, ULLRICH A AND MCGUIRE WL. (1987). Human breast cancer: correlation of relapse and survival with amplification of the Her-2/*neu* oncogene. *Science*, **235,** 177–182.

SMITH CS AND PILGRIM HI. (1971). Spontaneous neoplasms in germfree BALB/c Pi mice. *Proc. Soc. Exp. Biol. Med.*, **138,** 542.

SPARANO JA AND O'BOYLE K. (1992). The potential role for biological therapies in the treatment of breast cancer. *Semin. Oncol.*, **19,** 333–341.

SUDA Y, AIZAWA S, YASUHIDE F, YAGI T, IKAWA Y, SAITOH K, YAMADA Y, TOYOSHIMA K AND YAMAMOTO T. (1990). Induction of a variety of tumours by c-erb B2 and clonal nature of lymphomas even with the mutated gene (Val 659- Gln 659). *EMBO J.*, **9,** 181–190.

THOMAS H AND BALKWILL FR. (1994). Oncogene transgenic mice as therapeutic models in cancer research. *Eur. J. Cancer*, **30A,** 533–537.

VAN DE VIJVER MJ, VAN DE BERSSELAAR R, DEVILEE P, CORNELISSE C, PETERSE J AND NUSSE R. (1987). Amplification of the *neu* (c-erbB2) oncogene in human mammary tumours is relatively frequent and is often accompanied by amplification of the linked c-erbA oncogene. *Mol. Cell. Biol.*, **7,** 2019–2023.

VAN DE VIJVER MJ, PETERSE JL, MOOI WJ, WISMAN P, LOMANS J, DALESIO O AND NUSSE R. (1988). *Neu*-protein overexpression in breast cancer: association with comedo-type ductal carcinoma in situ and limited prognostic value in stage II breast cancer. *N. Engl. J. Med.*, **319,** 1239–1245.

VESSELINOVITCH SD, RAO KVN AND MIHAILOVICH N. (1975). Factors modulating benzidine carcinogenicity bioassay. *Cancer Res.*, **35,** 2814–2819.

YOKOTA J, YAMAMOTO T, TOYOSHIMA K, TERADA M, SUGIMURA T, BATTIFORA H AND CLINE MJ. (1986). Amplification of c-erbB2 oncogene in human adenocarcinomas *in vivo*. *Lancet*, **1,** 765.